# Clinical experiences with venetoclax and other pro-apoptotic agents in lymphoid malignancies: lessons from monotherapy and chemotherapy combination

**DOI:** 10.1186/s13045-022-01295-3

**Published:** 2022-06-03

**Authors:** Thomas E. Lew, John F. Seymour

**Affiliations:** 1 Department of Clinical Haematology, The Royal Melbourne Hospital and Peter MacCallum Cancer Centre, Melbourne, VIC 3000 Australia; 2 Blood Cells and Blood Cancer Division, Walter and Eliza Hall Institute of Medical Research, Parkville, Australia; 3 Faculty of Medicine, Dentistry and Health Sciences, The University of Melbourne, Parkville, Australia

**Keywords:** Apoptosis, Programmed cell death, BCL2, Venetoclax, Navitoclax, Lymphoma, Chronic lymphocytic leukemia, Non-Hodgkin lymphoma, Multiple myeloma

## Abstract

BH3-mimetics are a novel drug class of small molecule inhibitors of BCL2 family proteins which restore apoptosis in malignant cells. The only currently approved BH3-mimetic, the selective BCL2 inhibitor venetoclax, is highly efficacious in chronic lymphocytic leukemia and has rapidly advanced to an approved standard of care in frontline and relapsed disease in combination with anti-CD20 monoclonal antibodies. In this context, tumour lysis syndrome and myelosuppression are the most commonly encountered toxicities and are readily manageable with established protocols. Venetoclax is active in other lymphoid malignancies including several B cell non-Hodgkin lymphomas, acute lymphoblastic leukemia and multiple myeloma, with the highest intrinsic sensitivity observed in mantle cell lymphoma and Waldenstrom macroglobulinemia. Venetoclax combination with standard regimens in follicular lymphoma, multiple myeloma and aggressive B cell neoplasms has shown some promise, but further studies are required to optimize dose and scheduling to mitigate increased myelosuppression and infection risk, and to find validated biomarkers of venetoclax sensitivity. Future research will focus on overcoming venetoclax resistance, targeting other BCL2 family members and the rational design of synergistic combinations.

## Introduction

BCL2 and other components of the intrinsic cell death pathway are critical regulators of cell survival, frequently perturbed by cancer cells resulting in evasion of programmed cell death [1]. Initially identified as the fusion partner of the immunoglobulin heavy chain gene locus in the classic t(14;18) translocation of follicular lymphoma (FL), BCL2 overexpression was recognized to confer resistance to apoptosis [2]. This seminal observation instigated the ensuing decades of research that characterized the complex mechanisms of intrinsic cell death and lay the foundations for the development of therapeutics capable of restoring apoptosis in malignant cells. In brief, apoptosis is triggered when “pro-death/damage sensing” BH3-only proteins activate the downstream effector molecules BAX and BAK, which dimerize on the mitochondrial outer membrane surface and permeabilize it, triggering the release of cytochrome C, caspase activation and committing the cell to programmed death. BCL2 family proteins (BCL2, BCL-X_L_, MCL1, etc.) sequester BH3-only proteins and prevent apoptosis, and these anti-apoptotic properties are frequently upregulated in malignancy, making them an attractive therapeutic target [3].

**Fig. 1:**
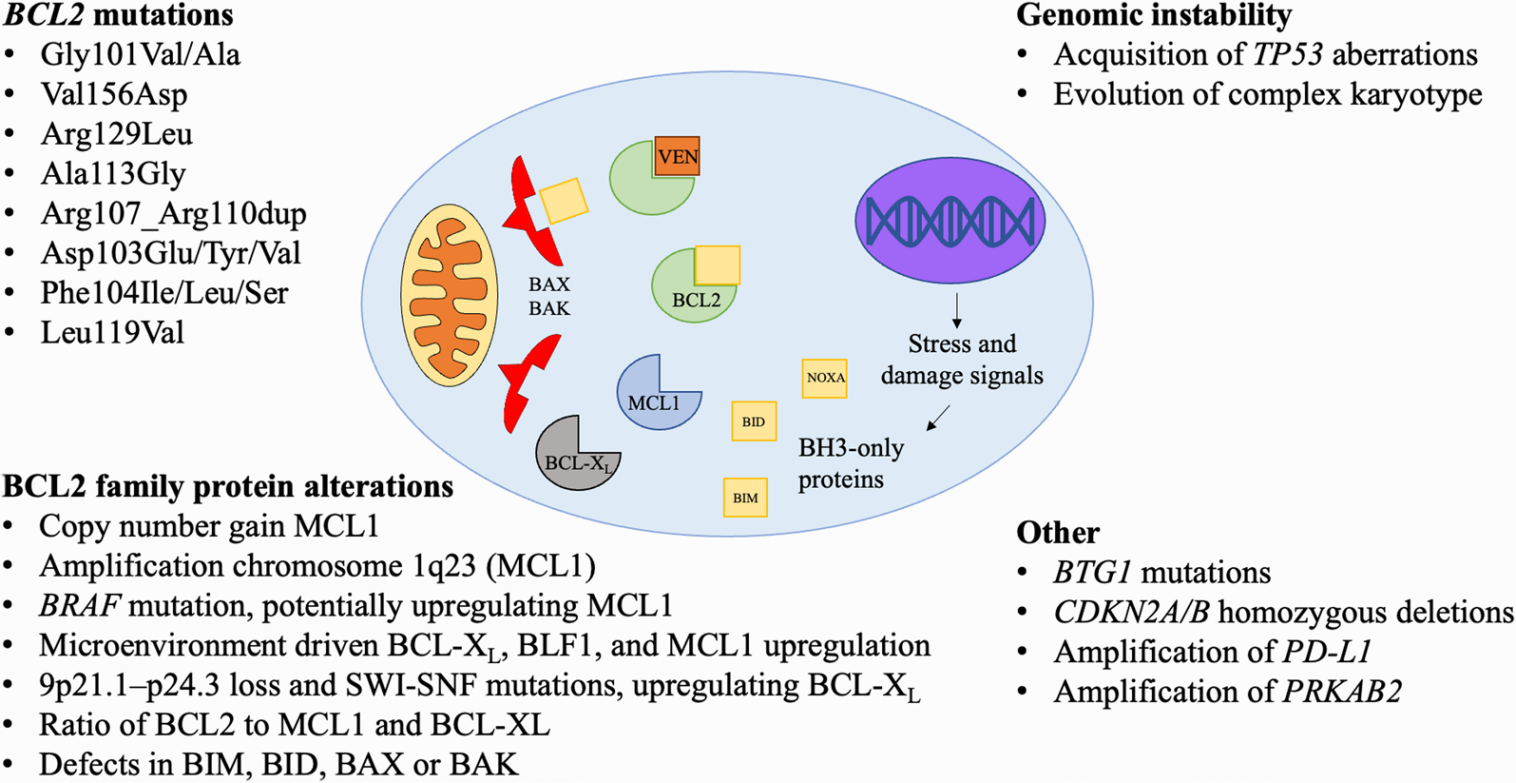
Mechanisms of venetoclax resistance in lymphoid malignancies

The first attempt to target BCL2 using the antisense oligonucleotide oblimersen (G3139) demonstrated limited clinical efficacy in chronic lymphocytic leukemia/small cell lymphoma (CLL/SLL) and B cell non-Hodgkin lymphoma (B-NHL) [4–6] and subsequent preclinical studies cast doubt over its capacity to effectively target BCL2 in tumor cells [7]. In contrast with oblimersen, BH3-mimetics are specific, potent and selective small molecule inhibitors of BCL2 family proteins, which disable their capacity to sequester “pro-death” BH3-only molecules. Released from the inhibitory action of BCL2 and related proteins, these pro-apoptotic BH3-only proteins are then able to activate BAX and BAK and initiate apoptosis (Fig. 1). BH3-mimetics are distinguished by their various selectivity for members of the BCL2 family (BCL2, BCL-X_L_, MCL1, etc.), and healthy and malignant cell types exhibit variable dependencies on these proteins to maintain survival [8].

The most clinically advanced and only licensed BH3-mimetic is venetoclax (ABT-199/GDC-0199), an orally bioavailable selective inhibitor of BCL2 [9]. In CLL/SLL, BCL2 is universally overexpressed and crucial to the evasion of cellular apoptosis [10], underpinning the potent clinical efficacy of venetoclax in patients with this disease and the first regulatory approval for a BH3-mimetic drug for this indication [11]. In contrast to inhibitors of B cell receptor signaling (BCRis), venetoclax frequently induces deep remissions and undetectable measurable residual disease (uMRD) status in patients with CLL/SLL, facilitating time-limited combination therapies [12, 13]. To date, venetoclax is approved for clinical use by the US Food and Drug Administration (FDA) and the European Medicines Agency (EMA) and regulatory authorities from many other countries for the management of relapsed and frontline CLL/SLL and acute myeloid leukemia (AML), with promising data in myeloma and several lymphoid malignancies (Table 1), with a notable exception of peripheral T cell lymphoma, where responses were rarely observed a in phase II study [14]. Another clinically advanced but currently unregistered BH3-mimetic is the predecessor molecule to venetoclax, the BCL2/BCL-X_L_/BCL-w inhibitor navitoclax (ABT-263), whose clinical efficacy in CLL/SLL was compromised by dose-limiting thrombocytopenia due to the reliance of platelets on BCL-X_L_ for survival [15, 16]. Other BH3-mimetics with reported clinical data include the pan-BCL2 family inhibitor obatoclax, which demonstrated limited selectivity for BCL2, minimal clinical efficacy and troublesome off-target neuropsychiatric toxicities in phase I trials [17, 18] and the MCL1 inhibitor AMG-176, which achieved infrequent responses (12%) in relapsed/refractory (R/R) multiple myeloma [19]. More recently, preliminary safety and efficacy data have been presented in abstract form from the first-in-human studies of two selective BCL2 inhibitors, BGB-11417 and lisaftoclax (APG-2575), in patients with CLL/SLL and B-NHLs [20–23]. Many other BH3-mimetic compounds are in the early phases of clinical development [24].

**Table 1:** Current US Food and Drug Administration (FDA) and European Medicines Agency (EMA) approvals for venetoclax

Indication	Regimen
*FDA*	
Treatment of patients with CLL with del(17p) who have been treated with at least one prior therapy [51]	Monotherapy
Treatment of patients with CLL who have received at least one prior therapy [32]	2 years fixed duration, combined with rituximab
Treatment of patients with previously untreated CLL [31]	1 year fixed duration, combined with obinutuzumab
Treatment of patients with newly diagnosed AML who are ≥ 75 years old or ineligible for intensive induction due to comorbidities [151, 152]	Combined with azacytidine, decitabine or lose-dose cytarabine
*EMA*	
Treatment of patients with CLL with del(17p) or *TP53* mutation who are unsuitable for or have failed a B cell receptor pathway inhibitor [51]	Monotherapy
Treatment of patients without del(17p) or *TP53* mutations who have failed both chemo-immunotherapy and a B cell receptor pathway inhibitor [55, 56]	Monotherapy
Treatment of patients with CLL who have received at least one prior therapy [32]	2 years fixed duration, combined with rituximab
Treatment of patients with previously untreated CLL [31]	1 year fixed duration, combined with obinutuzumab
Treatment of patients with newly diagnosed AML who are ineligible for intensive chemotherapy [151]	Combined with a hypomethylating agent

BH3-mimetics represent an exciting novel class of rationally designed and highly targeted therapeutics, capable of inducing rapid and deep remissions in CLL, AML and several other hematological malignancies. This review will principally focus on clinical data regarding the safety and efficacy of venetoclax and navitoclax in lymphoid neoplasms, and their combination with anti-CD20 monoclonal antibodies and conventional chemotherapy.

## Safety and toxicities of pro-apoptotic agents

The most significant adverse effects (AEs) of venetoclax and navitoclax are cytopenias and tumor lysis syndrome (TLS), although both can be readily prevented or treated in the majority of cases without significant clinical sequela. Because the earliest clinical use and approvals for venetoclax were in patients with CLL/SLL, the majority of the safety data available are derived from patients with this condition.

### Tumor lysis syndrome

Consistent with its mechanism of action of direct activation of apoptosis, clinically significant TLS is a well-described toxicity of venetoclax, most commonly seen in the context of CLL/SLL, and rarely in mantle cell lymphoma (MCL). During the dose ramp-up of the earliest phase I/Ib studies of venetoclax in patients with CLL/SLL, there were two fatalities due to clinical TLS and one instance of acute renal failure requiring dialysis [11, 25]. In response, the protocol was adapted for the expansion cohort of the phase I study, with an initial dose of 20 mg daily, weekly step-wise dose escalation and an aggressive TLS risk-adapted prophylaxis and monitoring program. Patients with any lymph node ≥ 10 cm or ≥ 5 cm with an absolute lymphocyte count (ALC) ≥ 25 × 10^9^/L are considered at high-risk for TLS, and inpatient monitoring, intravenous hydration and consideration of prophylactic rasburicase are recommended. Utilizing this strategy, there was only one case of laboratory TLS and no clinical TLS among the 60 patient expansion cohort of the phase I study [11]. The safety of this approach was further validated in a cohort of 350 patients receiving venetoclax for CLL, in which no patients met formal Howard criteria for laboratory or clinical TLS, although investigator assessed TLS and brief dose interruptions were required in a minority of patients, with all ultimately escalating to the recommended dose of 400 mg daily [26]. In addition to CLL/SLL disease burden, patients with impaired renal function are also at increased risk of TLS and warrant close attention. Drug–drug interactions that increase venetoclax levels, such as concomitant administration of potent CYP3A4 inhibitors, also potentiate TLS risk and should be avoided during dose ramp-up. Typically, TLS occurs within 6–24 h of venetoclax initiation or dose escalation [27]. As venetoclax is increasingly used in combination, many regimens are designed with a lead-in using anti-CD20 monoclonal antibodies or Bruton tyrosine kinase inhibitors (BTKis) which may reduce tumor bulk, reclassify the patient’s TLS risk and facilitate less intensive monitoring procedures [28, 29]. Despite this theoretical benefit, in a phase Ib study of venetoclax–obinutuzumab in which two alternative sequencing strategies were compared, laboratory TLS was uncommon, clinical TLS did not occur, and TLS rates did not differ between cohorts treated with venetoclax or obinutuzumab first, likely reflecting the effectiveness of the dose ramp-up strategy to mitigate the majority of TLS risk irrespective of sequencing [30]. In the phase III CLL14 study which defined the standard of care use of venetoclax in patients with previously untreated CLL/SLL and comorbidities or renal impairment, patients were randomized to one year of venetoclax plus obinutuzumab or chlorambucil plus obinutuzumab. In the trial design, patients received 3–4 doses of obinutuzumab prior to venetoclax, with three instances of laboratory TLS during the obinutuzumab run-in and no cases after commencement of venetoclax [31]. The phase III MURANO study defined the standard approach in patients with R/R CLL by randomizing patients to two years of venetoclax plus rituximab or bendamustine–rituximab (BR). In this study, rituximab commenced after the venetoclax dose ramp-up and grade III/IV TLS occurred in 3.1% of patients, with only one instance of clinical TLS (elevated creatinine) [32]. Overall, clinicians should be vigilant of the risk of TLS, but note that clinically significant sequela are rare with the careful application of the established ramp-up and risk-adapted prophylaxis strategy recommended in the product information [33]. When used in the frontline setting, clinicians should be cognizant that TLS can also occur during the obinutuzumab run-in prior to the initiation of venetoclax.

Outside the context of CLL/SLL, clinical TLS has been reported in patients receiving venetoclax for MCL, including one fatality. For this disease, an initial dose of 20 mg daily, weekly dose ramp-up and vigilant TLS prophylaxis and monitoring is strongly recommended [34]. For patients with less intrinsically sensitive disease such as FL and diffuse large B cell lymphoma (DLBCL), patients have been safely commenced at doses of 400 mg daily and up-titrated to 800-1200 mg daily with rare, manageable laboratory TLS and no instances of clinical TLS [35].

### Myeloid compartment toxicities and infections

Cytopenias are commonly observed in patients receiving venetoclax, particularly neutropenia, as BCL2 supports survival of granulocyte–macrophage precursor cells [36]. In a pooled analysis of 350 patients receiving venetoclax 400 mg daily as monotherapy for CLL, the most common grade III/IV toxicities were neutropenia (37%), anemia (17%) and thrombocytopenia (14%), although these most frequently emerged during dose ramp-up and the first 3 months of therapy, with a low rate of new onset severe cytopenias subsequently. Neutropenia was the most common reason for dose reductions or interruptions (required in 9% of patients), whereas this was rarely required for anemia or thrombocytopenia (< 2%). G-CSF support or brief dose pauses led to resolution of neutropenia in the majority of cases, and permanent discontinuation due to cytopenias was very rare (< 1%). Serious infections occurred in 15% of patients with grade III/IV neutropenia, and death due to infectious complications was rare (~ 1%). Opportunistic infections such as *Pneumocystis jiroveci* pneumonia, invasive fungal diseases or herpetic eruptions are very uncommon, even in among heavily pre-treated patients (3.1%), and no specific prophylaxis is generally warranted [26].

Consistent with these observations in the monotherapy setting, grade III/IV neutropenia was commonly observed in the MURANO trial. Although grade III/IV neutropenia occurred more frequently in the venetoclax–rituximab arm than with BR (58% vs 39%), febrile neutropenia was uncommon (4% vs 10%), and rates of infections and infestations were comparable (18% vs 22%)[32]. Similarly, in the CLL14 study, grade III/IV neutropenia occurred in 53% of the venetoclax–obinutuzumab and 48% in the chlorambucil–obinutuzumab arm and grade III/IV infections occurred in 18% and 15%, respectively [31]. In summary, clinicians should be conscious that neutropenia is a frequent toxicity of venetoclax; however, the use of G-CSF and/or a treatment pause in persistent cases is typically effective, such that premature discontinuation of therapy due to cytopenias is rarely if ever appropriate and can adversely impact therapeutic benefit [37]. Although infectious sequela can occur, these are comparable to rates seen with chemo-immunotherapy regimens. Outside of CLL/SLL, cytopenias were less frequently reported among similarly heavily pre-treated cohorts of patients with MCL, FL and DLBCL (grade III/IV anemia 15%, neutropenia 11%) [35, 38].

A recent report of patients treated with longer-term continuous venetoclax for heavily pre-treated CLL/SLL found that 28% of patients experienced persistent cytopenias (≥ 4 months), with 80% of such patients demonstrating clonal myeloid abnormalities. Although treatment-related myeloid neoplasms (tMNs) were described, these exclusively occurred in the setting of prior fludarabine exposure at rates comparable to historical cohorts. Interestingly, mutations of the intrinsic death pathway effector *BAX* were identified in the myeloid compartment in 32% of evaluable patients with a low burden of bone marrow (BM) CLL. Although these mutations conferred resistance to venetoclax, they were not clearly associated with the emergence of myeloid neoplasms [39]. These findings are of uncertain relevance to patients receiving time-limited venetoclax, but nevertheless demonstrate that chronic BH3-mimetic therapy can exert on-target survival pressures and clonal selection within the non-malignant myeloid compartment. In patients with persistent cytopenias on venetoclax, especially those with prior fludarabine exposure, BM evaluation, cytogenetics and molecular studies are recommended to exclude tMN.

### Gastrointestinal toxicities

Most patients (75%) will experience transient gastrointestinal AEs on venetoclax, typically mild diarrhea or nausea. Grade III/IV gastrointestinal symptoms are rare and typically transient (median duration 5 days) at 400 mg but become more troublesome at higher doses. Most incidences emerge during dose ramp-up and resolve over time, although a small subset of patients will have persistent mild symptoms [26]. After infectious or other clinically suspected etiologies are excluded, supportive care measures such as anti-emetics and/or anti-motility agents are generally effective and facilitate ongoing treatment in the majority of cases [27]. Alternative drug formulations are being explored that may improve gastrointestinal tolerance [40, 41].

### Thrombocytopenia and navitoclax

The precursor to venetoclax, navitoclax, is a small molecule BCL2 inhibitor that exhibits a concomitant 200-fold inhibitory action on BCL-X_L_ and BCL-w. The toxicity profile to navitoclax is similar to venetoclax, with frequent neutropenia, uncommon infectious complications and transient, typically low-grade, gastrointestinal symptoms. However, navitoclax additionally causes dose-related acute thrombocytopenia due to the reliance of platelets on BCL-X_L_ for survival [16], with nadirs 2–5 days after dose initiation, partial recovery during ongoing dosing and full resolution with drug cessation. Grade III/IV thrombocytopenia occurred in 30–50% of patients receiving navitoclax in early phase trials, although clinically significant bleeding has only been rarely reported [42–46], perhaps due to the greater susceptibility of older platelets to apoptosis and persistence of younger, more hemostatically effective platelets. Due to this dose-limiting toxicity, the clinical development of navitoclax was de-prioritized in favor of venetoclax, whose selectivity of BCL2 preserved platelets [15, 42]. Therefore, the anti-neoplastic efficacy of navitoclax has not been studied at doses likely required to optimally inhibit BCL2 or to determine the potential anti-tumor effect of potent BCL-X_L_ inhibition. Partially due to this, TLS with navitoclax has also been observed in only a small proportion of patients [15, 42]. Nevertheless, the clinical potential of targeting BCL-X_L_ remains an area of significant interest, as BCL-X_L_ upregulation has been implicated in venetoclax resistance and responses to navitoclax have been observed in a range of lymphoid malignancies (summarized in Table 2)[47, 48]. Alternative scheduling or novel drug formulations may enable safe delivery of BCL-X_L_ inhibitors and reveal their true therapeutic potential [49, 50].

**Table 2:** Clinical trials of navitoclax in hematological malignancies

Study	Cohort	Design	ORR/CRR MRD	PFS/OS	III/IV toxicity (> 10%)	Note
*Phase I*						
Wilson et al.[45] NCT00406809	*n* = 55 R/R lymphoid malignancies	Dose escalation of navitoclax given 14 or 21 days out of 21-day cycle	ORR 22%	Median PFS 16 months (among responders)	Neutropenia 31% Thrombocytopenia 53%	Continuous dosing appeared to reduce platelet nadir
Roberts et al.[15] NCT00481091	*n* = 29 RR CLL	Dose escalation of navitoclax given 14 or 21 days out of 21-day cycle	ORR 35% among patients receiving ≥ 110 mg daily	Median PFS 25 months	Neutropenia 28% Thrombocytopenia 28%	Navitoclax MTD: 250 mg daily
Roberts et al.[43] NCT00788684	*n* = 29 Previously treated B cell malignancies FL (*n* = 12) Aggressive lymphoma (*n* = 9) CLL/SLL (*n* = 5)	3 + 3 dose escalation of navitoclax 200-325 mg daily + rituximabx4	FL: ORR 75% CRR 42% Aggressive lymphoma: ORR 11% CRR 11% CLL: ORR 100% CRR 0%	Median PFS 11 months	Neutropenia 28% Thrombocytopenia 17%	Navitoclax MTD: 250 mg daily
Pullarkat et al.[50] NCT03181126	*n* = 47 Pediatric and adult R/R ALL	Dose escalation of low dose navitoclax with venetoclax 400 mg daily, plus chemotherapy	ORR 66% CRR 60% uMRD 34%	Median DOR 4.2 months Median OS 7.8 months 28% proceeded to CAR-T or alloSCT	Febrile neutropenia 47% Neutropenia 38% Thrombocytopenia 26%	RP2D of navitoclax 50 mg daily for adult, 25 mg if < 45 kg, with venetoclax 400 mg daily and chemotherapy
*Phase II*						
Kipps et al.[42] NCT01087151	*n* = 118 Previously untreated CLL/SLL	Randomized 1:1:1 A: Rituximab × 8 B: Rituximab × 8 + navitoclax 12 weeks C: Rituximab × 8 + indefinite venetoclax Navitoclax 100 mg daily for 1^st^ week, then 250 mg daily	A: ORR 35% CRR 0% B: ORR 55% CRR 0% C: ORR 75% CRR 5%	A: Median PFS 9.1 months B: Median PFS 15.6 months C: Not reached	Arm C Neutropenia 45% Thrombocytopenia 33% Liver enzyme increase 25%	Study closed prematurely due to sponsor decision to develop ABT-199 (venetoclax)
de Vos et al.[46] NCT00406809	*N* = 26 Previously treated B cell malignancies A: FL (*n* = 11) B: Other (*n* = 15)	Navitoclax 150 mg daily for 1^st^ week, then 250 mg daily. Optional escalation to 325 mg daily if well tolerated. Continuous dosing	A: ORR 9% CRR 9% B: ORR 33% CRR 0%	Median PFS 4.9 months Median OS 24.8 months	Thrombocytopenia 39% Neutropenia 31%	

## Efficacy of venetoclax in chronic lymphocytic leukemia/small cell lymphoma

### Phase I/II studies

Published clinical trial data for venetoclax in CLL are summarized in Table 3. In the first-in-human and expansion cohorts of the phase I study, venetoclax achieved an objective response rate (ORR) of 79%, including complete responses (CRs) in 20% and undetectable BM disease by flow cytometry in 5%. These results were particularly encouraging given the study cohort was enriched for high-risk features including multiple prior therapies, *TP53* aberrations, fludarabine refractoriness and IGHV-unmutated status. Indeed, venetoclax achieved consistent response rates and deep remission across these high-risk subsets [11]. The efficacy of venetoclax in high-risk disease was formally evaluated in a subsequent phase II study of 158 patients with CLL/SLL harboring del(17p), where high response rates (ORR 86%) and frequent deep remissions (CR rate [CRR] 53%) were again demonstrated. Furthermore, uMRD by flow cytometry (defined as < 1 CLL cell per 10,000 leucocytes, when ≥ 200,000 leukocytes are analyzed) was observed in the peripheral blood (PB) and BM of 30% and 13%, respectively [51, 52]. These observations were in distinct contrast to the other highly effective targeted agents in CLL, namely BCRis such as ibrutinib and idelalisib, where durable remissions can be achieved for patients with high-risk disease, but deep remissions are rare and indefinite therapy is required [53, 54]. Venetoclax monotherapy has also demonstrated efficacy in the context of BCRi resistance or intolerance [55, 56], although refractoriness to BCRis is associated with inferior response rates and disease control after venetoclax on multivariate analyses [44]. Venetoclax monotherapy was also associated significantly improved quality of life among patients with R/R CLL in the single-arm VENICE II study [57].

**Table 3:** Clinical trials of venetoclax with or without anti-CD20 antibodies or chemotherapy in CLL/SLL

Study	Cohort	Design	ORR/CRR MRD	PFS/OS	III/IV toxicity (> 10%)	Note	
*Phase I/II*							
Roberts et al.[11] NCT01328626	R/R Dose finding: *n* = 56 Expansion: *n* = 60	Phase I 150-1200 mg venetoclax daily* 400 mg venetoclax daily*	ORR 79% CRR20% BM flow cytometry-neg 5%	15-month PFS 66% 24-month OS 84%	3 cases clinical TLS, one fatal Neutropenia 41% Anemia 12% Thrombocytopenia 12%		
Stilgenbauer et al.[51, 52] NCT01889186	del(17p) R/R: *n* = 158 TN: *n* = 5	Phase II 400 mg venetoclax daily*	ORR 77% CRR 20% PB uMRD 30% BM uMRD 13%	24-month PFS 54% 24-month OS 73%	No clinical TLS Neutropenia 40% Anemia 15% Thrombocytopenia 15% Pneumonia 10%		
Seymour et al.; Ma et al.[25, 58] NCT01682616	R/R *n* = 49	Phase Ib 200-400 mg venetoclax daily^ + rituximab × 6	ORR 86% CRR 53% BM uMRD 61%	5-year PFS 56% 5-year OS 86%	2 cases clinical TLS, one fatal Neutropenia 53% Anemia 14% Thrombocytopenia 16% Febrile neutropenia 12% Infections and infestations 16%	Among 33 patients with deep remission (CR or uMRD), 14 continued venetoclax and 19 ceased with similar PFS	
Coutre et al.[55] NCT02141282	Idelalisib exposed *n* = 36	Phase II 400 mg venetoclax daily*	ORR 67% CRR 8% PB uMRD 22% BM uMRD 6%	12-month PFS 79% 12-month OS 94%	No clinical TLS Neutropenia 50% Thrombocytopenia 25% Anemia 17%		
Jones et al.[56] NCT02141282	Ibrutinib exposed *n* = 91	Phase II 400 mg venetoclax daily*	ORR 65% CRR 9% PM uMRD 26% BM uMRD 5%	Median PFS 25 months 12-month OS 92%	2 cases laboratory TLS Neutropenia 51% Anemia 29% Thrombocytopenia 29% Lymphopenia 1% Febrile neutropenia 11%		
Flinn et al.[30] NCT01685892	R/R: *n* = 50 TN: *n* = 32	Phase Ib 100-400 mg venetoclax daily ^&^ + obinutuzumab × 6	R/R ORR 95% CRR 37% PM uMRD 64% BM uMRD 62% TN ORR 100% CRR 78% PM uMRD 91% BM uMRD 78%	R/R 24-month PFS 85% OS NA TN 24-month PFS 91% OS NA	Neutropenia 53–58% Thrombocytopenia 22% Infection 13–29%		
Cochrane et al.[57] NCT02980731	R/R *n* = 210	Phase IIIb 400 mg venetoclax daily^#^	ORR 77% CRR 19% QoL + 9.3 points by EORTC QLQ-C30	12-month PFS 83% 12-month OS 88%	Neutropenia 32% Thrombocytopenia 17% Anemia 11%		
*Phase III*							
MURANO [32, 62–64] NCT02005471	R/R Venetoclax + rituximab *n* = 194	400 mg venetoclax daily 24 months + rituximab × 6^+^	ORR 93% CRR 27% PB uMRD 62% BM uMRD 27%	Median PFS 54 months 5-year OS 82%	TLS 3%, 1 case clinical TLS Neutropenia 58% Infection 18%		
	Bendamustine + rituximab *n* = 195	70 mg/m^2^ bendamustine D1-2 Q28D × 6 + rituximab × 6		ORR 68% CRR 8% PB uMRD 13% BM uMRD 2%	Median PFS 17 months 5-year OS 62%	TLS 1%, 1 case clinical TLS Neutropenia 39% Infection 22%	
CLL14 [28, 31, 65] NCT02242942	TN CIRS > 6 or CrCl < 70 Venetoclax + obinutuzumab *n* = 216	400 mg venetoclax daily 12 months + obinutuzumab × 6 cycles	ORR 85% CRR 50% PB uMRD 76% BM uMRD 57%	3-year PFS 82% 24-month OS 92%	3 cases lab TLS Neutropenia 53% Infection 18%		
	Chlorambucil + obinutuzumab *n* = 216	0.5 mg/kg chlorambucil D1 + D15 Q28D × 12 + obinutuzumab × 6 cycles	ORR 71% CRR 23% PB uMRD 35% BM uMRD 17%	3-year PFS 50% 24-month OS 93%	5 cases lab TLS Neutropenia 48% Infection 15%		
CLL13 (GAIA) [68] NCT02950051	TN CIRS ≤ 6 and CrCl > 70 Fludarabine, cyclophosphamide + rituximab (≤ 65y) or bendamustine + rituximab (> 65y) *n* = 216	25 mg/m^2^ fludarabine D1-3, 250 mg/m^2^ cyclophosphamide D1-3 + rituximab Q28Dx6; 90 mg/m^2^ bendamustine D1-2 Q28D × 6 + rituximab × 6	Month 15 ORR 81% CRR 31% PB uMRD 52% BM uMRD 37%	NA	Neutropenia 52% Infections 20% Febrile neutropenia 11% Thrombocytopenia 10%		
	Venetoclax + rituximab *n* = *237*	400 mg venetoclax daily 12 months + rituximab	Month 15 ORR 93% CRR 49% PB uMRD 57% BM uMRD 43%	NA	Neutropenia 46% Infections 11% TLS 10%		
	Venetoclax + obinutuzumab *n* = 228	400 mg venetoclax daily 12 months + obinutuzumab × 6 cycles	Month 15 ORR 96% CRR 57% PB uMRD 87% BM uMRD 73%	NA	Neutropenia 59% Thrombocytopenia 18% Infections 14% Infusion reaction 11%		
	Venetoclax + ibrutinib + obinutuzumab *n* = 231	400 mg venetoclax daily 12 months + obinutuzumab × 6 cycles + ibrutinib 420 mg daily to 12 months if uMRD, for 36 months if MRD +	Month 15 ORR 94% CRR 62% PB uMRD 92% BM uMRD 78%	NA	Neutropenia 57% Infections 22% Thrombocytopenia 16%		
*Venetoclax plus chemotherapy*							
CLL2-BAG [59] NCT02401503	*n* = 63 TN: *n* = 34 R/R: *n* = 29	De-bulking *if tumor bulk* (ALC > 25,000/uL or adenopathy ≥ 5 cm): 70 mg/m^2^ bendamustine D1-2 Q28D × 2 Induction: Venetoclax + obinutuzumab (2 cycles) Maintenance: Venetoclax up to 24 months + obinutuzumab	TN: ORR 100% CRR 50% PB uMRD 91% RR: ORR 90% CRR 28% PB uMRD 83%	TN: 15-month PFS 100% 15-month OS 100% RR: 15-month PFS 92% 15-month OS 95%	Neutropenia 44% Infections 14% Thrombocytopenia 13% Anemia 11%		
Lipsky et al.[153] NCT03609593	TN *n* = 26	50-90 mg/m^2^ bendamustine D1-2 Q28D + rituximab × 3 *followed by* 400 mg venetoclax daily 12 months + rituximab × 6	ORR 100% CRR 92% PB uMRD 100% BM uMRD 90%	NA	Neutropenia 11%	95% of patients with medium–high TLS risk were risk de-escalated after de-bulking chemotherapy	
Flinn et al. [60] NCT03406156	TN, no del(17p), medium–high TLS risk *n* = 120	Obinutuzumab ± bendamustine for up to 6 cycles, *followed by* venetoclax–obinutuzumab if low-risk TLS status achieved	ORR 90% CRR 36% PB uMRD 89%	18 months PFS 94%	Neutropenia 28–41%		

CIRS = Cumulative Illness Rating Scale, CrCl = creatinine clearance, *continuous therapy until progression, death or other reason to withdraw from trial, ^continuous venetoclax therapy, cessation permitted in good response at clinician discretion, & = continuous venetoclax for patients with relapse and refractory disease, fixed-duration therapy for 12 months in treatment-naïve patients with optional additional 12 months of therapy if not in CR or BM MRD-neg response, #continuous therapy until progression, unacceptable toxicity or two years total therapy. Patients in countries without commercial access to venetoclax could continue for a further two years if ongoing clinical benefit. QoL = quality of life. EORTC QLQ-C30 = European Organization for Research and Treatment of Cancer Quality of Life Questionnaire Core 30

Supported by in vitro and clinical evidence of synergy between anti-CD20 monoclonal antibodies and BH3-mimetics [9, 42, 43], 49 patients were enrolled in a phase Ib study of venetoclax plus rituximab, achieving frequent and deep responses (ORR 86% CRR 53%), with a remarkable frequency of BM uMRD in a R/R cohort (61%). Hematological toxicities predominated, with no unexpected AEs observed with the combination regimen [25]. At latest follow-up, the 5-year estimated progression-free survival (PFS) and overall survival (OS) rates were 56% and 86%. Among 33 patients who attained CR or uMRD, 14 elected to continue venetoclax monotherapy and 19 ceased, with comparable PFS between the two groups (ongoing response at 5 years in 71% for ongoing therapy and 79% for fixed duration) [58]. Although non-randomized, these results suggest that venetoclax can be ceased among patients in deep response without compromising durability of disease control, with the potential advantages of minimizing toxicity, cost and the selection pressure for venetoclax-resistant subclones. A phase Ib study of venetoclax–obinutuzumab has also demonstrated responses in 95–100% of patients, with rates of BM uMRD ranging from 62–78% across treatment-naïve and R/R cohorts [30]. Although a formal randomized comparison of venetoclax with or without anti-CD20 antibodies has not been performed and cohort characteristics differ significantly between monotherapy and combination studies, these datasets and other non-randomized comparisons suggest that depth of remission is substantially increased through combination, without increase in clinically serious AEs [12]. Overall, early phase clinical trials demonstrated frequent, deep and durable remissions were achievable with venetoclax, including patients with biologically adverse disease. Furthermore, observation that uMRD could be attained in the majority of patients with durable treatment-free remissions led to the preferential development of the fixed-duration regimens that were evaluated in phase III trials and defined current standard of care approaches.

### Combining venetoclax with conventional chemotherapy in CLL/SLL

The potent single-agent efficacy of venetoclax in CLL/SLL and frequent neutropenia somewhat dissuade combination with chemotherapy, which may add little additional efficacy and likely synergistic myelosuppression. Nevertheless, the phase II CLL2-BAG study investigated the use of bendamustine for two cycles to de-bulk disease in patients with high tumor volume, followed by obinutuzumab and venetoclax for up to 2 years. Of the patients who received de-bulking chemotherapy (31 treatment-naïve; 14 R/R), 53% had responsive disease, approximately one-third of patients experienced grade III/IV AEs and the majority of patients with high TLS risk due to ALC or bulky adenopathy had resolution of these high-risk features prior to venetoclax. Response rates, survival outcomes and toxicities were comparable to other anti-CD20 combination regimens [59]. In a recent preliminary report from a larger phase IIIb study of de-bulking obinutuzumab ± bendamustine with TLS risk re-evaluation after every 2 cycles followed by venetoclax–obinutuzumab once low-TLS risk status was achieved, 92% of patients attained low-risk status after de-bulking. However, grade III/IV toxicities occurred in 72–76% of patients, including serious AEs in 18–24% (most commonly pneumonia and COVID-19) [60]. Given the low incidence of clinical TLS with venetoclax using standard protocols, the utility of de-bulking chemotherapy is unclear and may increase short and longer-term toxicities. The availability of other potent targeted agents with evidence of synergistic efficacy and non-overlapping toxicities has focused research efforts on these “chemotherapy-free” combination regimens [29, 61].

### Phase III studies

For patients with R/R disease, the MURANO study randomized subjects to receive venetoclax for 2 years after completion of the 5-week dose ramp-up combined with six doses of rituximab or BR for six cycles. Patients receiving venetoclax–rituximab demonstrated superior response rates, response depth, PFS and OS. At most recent follow-up, the median PFS after venetoclax–rituximab was 54 months, with an estimated OS of 82% at 5 years. PB uMRD was achieved in 62% of patents, and ~ 40% of patients with PB uMRD at the end of fixed-duration venetoclax treatment maintained ongoing uMRD after 3 years off therapy, demonstrating that deep treatment-free remissions can be sustained after time-limited combination therapy [32, 62–64].

The CLL14 trial randomized treatment-naïve patients with comorbidities (Cumulative Illness Rating Score [CIRS] > 6) or renal impairment (creatinine clearance [CrCl] < 70 ml/min) to 12 months of venetoclax or chlorambucil, each combined with 6 cycles of obinutuzumab. The venetoclax regimen demonstrated superior response depth and PFS with comparable toxicity and OS [31]. In the venetoclax–obinutuzumab cohort, 76% of patients attained PB uMRD and the estimated 3-year PFS was 82% [28, 65]. In both MURANO and CLL14, superiority of the venetoclax-based regimen was observed across subgroups, with greatest benefit among patients with del(17p), *TP53* mutations and IGHV-unmutated status, for whom outcomes after conventional chemo-immunotherapy are inferior [66]. Whether a subset of patients may benefit from more prolonged venetoclax treatment remains controversial, although deepening of response beyond two years of therapy is rare [12]. To date, randomized data are limited to support the use of venetoclax-based regimens for treatment-naïve fit patients, in distinction to ibrutinib which has demonstrated superiority to the standard chemo-immunotherapy regimen FCR (fludarabine, cyclophosphamide, rituximab)[67]. Preliminary response/MRD data from the CLL13 study have recently been reported, in which fit patients (CIRS ≤ 6 and CrCl < 70) were randomized to chemo-immunotherapy (FCR if ≤ 65/BR if > 65) vs venetoclax–rituximab vs venetoclax–obinutuzumab vs obinutuzumab–ibrutinib–venetoclax. At 15 months, PB uMRD rates were significantly higher in the venetoclax–obinutuzumab (87%) and obinutuzumab–ibrutinib–venetoclax group (92%) compared to chemo-immunotherapy (52%), whereas PB uMRD rates after venetoclax–rituximab were similar (57%). Toxicities were similar across arms, and although differences in uMRD attainment are likely be reflected in PFS and OS, these outcomes are yet to be formally reported [68]. While these survival data and regulatory considerations are awaited, access to frontline venetoclax-based regimens in patients otherwise fit for intensive chemo-immunotherapy will be limited in some countries. Nevertheless, venetoclax- or BTKi-based regimens are recommended over chemo-immunotherapy for fit patients with disease harboring *TP53* aberrations, for whom durable remissions are not achieved with FCR. For fit patients with p53 wild-type, IGHV-mutated disease, FCR achieves very durable remissions and likely cures in ~ 50–60%, and best frontline management for this subgroup will likely remain controversial for some time [69].

### Venetoclax re-treatment

Emerging evidence suggests that many patients who cease venetoclax in deep response may retain sensitivity to BCL2 inhibition at disease relapse. This is congruent with the observation that resistance mutations are typically observed only after prolonged exposure and have not been detected among patients treated with time-limited venetoclax-based regimens to date [48]. After a median time off therapy of 3.2 years, six patients (32%) who ceased in deep response in the phase Ib study of venetoclax–rituximab have had progressive disease (PD), and four have been re-treated with venetoclax–rituximab. All had ceased drug for > 24 months, and all had responsive disease, with second remissions ranging from 19 to 43 months (three ongoing) [58]. In two separate preliminary reports each with 18 response-evaluable patients with similar results, venetoclax-based re-treatment achieved responses in 72% of patients, including CRs and remissions beyond one year [70, 71]. Although longer-term follow-up of larger cohorts is required, these data suggest that re-treatment is effective for some patients with PD after time-limited therapy. Further study of clinicopathological predictors of successful re-treatment is warranted to maximize the therapeutic benefit of this strategy [72, 73]. Given the dismal prognosis of patients who are resistant to both venetoclax and BTKis [74], venetoclax re-treatment may become an attractive option, prolonging the clinical benefit of this therapeutic modality. A prospective phase II study of venetoclax–obinutuzumab re-treatment for patients with CLL relapsing > 1 year after previous time-limited venetoclax–obinutuzumab is in progress (ReVenG: NCT 04895436)[75].

## Efficacy of venetoclax in other B cell neoplasms

### Mantle cell lymphoma

BCL2 is frequently upregulated in MCL, driven by defective degradation by the E3 ubiquitin ligase FBXO10 and increased transcription through BTK signaling driven activation of the nuclear factor-kappa B pathway [76]. BCL2 inhibition potently induces apoptosis in cell lines and primary MCL cells, especially in cells with a high BCL2-to-MCL1 ratio [77]. These preclinical observations made BCL2 inhibition an attractive strategy warranting clinical trials in patients with MCL (summarized in Table 4).

**Table 4:** Studies of venetoclax with or without anti-CD20 antibodies or chemotherapy in B-NHL

Study	Cohort	Design	ORR/CRR MRD	PFS/OS	III/IV toxicity (> 10%)	Note
*Mantle cell lymphoma*						
Davids et al.[35, 38] NCT01328626	*n* = 28 R/R MCL (median 3 prior lines of therapy) Lenalidomide and ibrutinib naïve 18% bortezomib exposed	Phase 1 dose escalation venetoclax monotherapy 200-1200 mg daily	ORR 75% CRR 21%	Median PFS 11 months Median DOR 16 months 12-month OS: 82%	Overall cohort Anemia 15% Neutropenia 11%	RP2D 800 mg
Eyre et al.[79]	*n* = 20 R/R MCL (median 3 prior lines of therapy) BTKi exposed (90% resistant)	Retrospective analysis of venetoclax at doses of 200-1200 mg daily	ORR 53% CRR 18%	Median PFS 3 months Median OS 9 months	Pneumonia 15%	
Zhao et al.[80]	*n* = 24 R/R MCL (median 5 prior lines of therapy) BTKi resistant: 67%	Retrospective analysis of venetoclax-based regimens: Monotherapy: *n* = 12 + anti-CD20 antibody: *n* = 8 + BTKi: *n* = 3 + chemotherapy: *n* = 1	ORR 50% CRR 21%	Median PFS 8 months Median OS 13.5 months	NA	
Sawalha et al.[81]	*n* = 81 R/R MCL (median 3 prior lines of therapy)	Retrospective analysis of venetoclax-based regimens: Monotherapy: *n* = 50 + anti-CD20 antibody: *n* = 11 + BTKi: *n* = 16 + other: *n* = 4	ORR 42% CRR 18%	Median duration of venetoclax treatment 3 months Median OS 13 months	NA	
*Follicular lymphoma*						
Davids et al.[35, 38] NCT01328626	*n* = 29 R/R FL	Phase 1 dose escalation venetoclax monotherapy 200-1200 mg daily	ORR 38% CRR 17%	Median PFS 11 months Median DOR 27 months 12-month OS: 100%	Overall cohort Anemia 15% Neutropenia 11%	RP2D 1200 mg
de Vos et al.[86] NCT01594229	*n* = 32 R/R FL	Phase 1b dose escalation of venetoclax 50-1200 mg daily plus bendamustine–rutiximabx6	ORR 75% CR 38%	Median PFS and OS not reached	Overall cohort Neutropenia 60% Lymphopenia 38%	RP2D 800 mg in combination with bendamustine–rituximab
CAVALLI Ib [87] NCT02055820	*n* = 24 R/R FL: *n* = 4 TN FL: *n* = 20	Phase 1b dose escalation of venetoclax 200-800 mg in combination with R/G-CHOP	ORR 83% CRR 75%	12-month PFS 100% Ven-R-CHOP and 90% Ven-G-CHOP	Overall cohort Neutropenia 54% Febrile neutropenia 33% Thrombocytopenia 17% Anemia 13%	RP2D 800 mg C1D4-10 and D1-10 for subsequent cycles
Stathis et al.[84] NCT02877550	*n* = 25 TN FL	Phase I trial of venetoclax 600-800 mg daily plus obinutuzumab for 6 cycles, followed by obinutuzumab maintenance [2 years]	ORR 88% CRR 68%	12-month PFS 77%	Neutropenia 28%	RP2D 800 mg in combination with obinutuzumab
PrECOG 0403 [89] NCT03113422	*n* = 56 TN FL with high tumor burden	Phase II trial of venetoclax 800 mg plus bendamustine–rituximabx6	CRR 73%	24-month PFS 86% 24-month OS 94%	Neutropenia 16% Thrombocytopenia 14% TLS 14%	Opportunistic infections, combination considered unacceptably immunosuppressive
Zinzani et al.[90] NCT02187861	*n* = 163 R/R FL Arm A: *n* = 52 Arm B: *n* = 51 Arm C: *n* = 51 Safety run-in: *n* = 9	Phase II study of: Arm A: Venetoclax 800 mg daily for 1 year plus rituximab C1D1,8,15,22 & D1 of C4, C6, C8, C12 Arm B: Venetoclax 800 mg daily for 1 year plus bendamustine–rutiximabx6 Arm C: Bendamustine–rituximabx6	Arm A: ORR 35% CRR 17% Arm B: ORR 84% CRR 75% Arm C: ORR 84% CRR 69%	18-month PFS Arm A: 27% Arm B: 62% Arm C:59%	Arm A: Neutropenia 25% Arm B: Neutropenia 59% Thrombocytopenia 45% Anemia 14% Febrile neutropenia 12% Arm C: Neutropenia 28%	
*Diffuse large B cell lymphoma and aggressive B cell lymphomas*						
Davids et al.[35, 38] NCT01328626	*n* = 34 R/R DLBCL and PMBCL [2 patients]	Phase 1 dose escalation venetoclax monotherapy 200-1200 mg daily	ORR 18% CRR 12%	Median PFS 1 month 12-month OS: 32%	Overall cohort Anemia 15% Neutropenia 11%	RP2D 1200 mg
de Vos et al.[86] NCT01594229	*n* = 22 R/R DLBCL	Phase 1b dose escalation of venetoclax 50-1200 mg daily plus bendamustine–rutiximabx6	ORR 41% CR 14%	Median PFS 4 months Median OS 15 months	Overall cohort Neutropenia 60% Lymphopenia 38% Thrombocytopenia 28% Anemia 17%	RP2D 800 mg in combination with bendamustine–rituximab
CAVALLI 1b [87] NCT02055820	*n* = 18 TN DLBCL	Phase 1b dose escalation of venetoclax 200-800 mg in combination with R/G-CHOP	ORR 89% CRR 89%	12-month PFS 70% Ven-R-CHOP and 100% Ven-G-CHOP	Overall cohort Neutropenia 54% Febrile neutropenia 33% Thrombocytopenia 17% Anemia 13%	RP2D 800 mg C1D4-10 and D1-10 for subsequent cycles
Rutherford et al.[154] NCT03036904	*n* = 30 TN aggressive B-NHL DHL: *n* = 15 DLBCL: *n* = 9 Transformed NHL: *n* = 2 HGBCL-NOS: *n* = 2 PMBCL: *n* = 2	Phase 1 dose escalation of venetoclax 400-800 mg in combination with dose-adjusted-R-EPOCH	ORR 97% CRR 93%	24-month PFS: 83% 24-month OS: 90%	Neutropenia 83% Thrombocytopenia 70% Febrile neutropenia 63% Anemia 60% Serious gastrointestinal AEs 27%	RP2D 600 mg for 5 days per cycle
CAVALLI phase II [93] NCT02055820	*n* = 206 TN DLBCL IPI 2–5	Phase II study of venetoclax-Rx8 plus CHOPx6-8	ORR 83% CRR 69%	24-month PFS: 80% 24-month OS: 86%	Neutropenia 68% Febrile neutropenia 31% Infections 23% Anemia 24% Thrombocytopenia 22% Leukopenia 10%	Improved PFS compared to historical R-CHOP control (GOYA), esp if BCL2 + by IHC
ALLIANCE A051701 [95] NCT03984448	TN double-hit lymphoma (by FISH or expression) *n* = 36	Phase II/III randomized study DA-R-EPOCH	ORR 73% CRR 67%	Median PFS and OS not reached (median follow-up 7 months)	Neutropenia 67% Febrile neutropenia 36% Sepsis 14%	Early discontinuation due to excess deaths in DA-R-EPOCH + venetoclax arm
	*n* = *73*	DA-R-EPOCH + venetoclax 600 mg C1D4-8 and C2-6D1-5	ORR 58% CRR 50%	Median PFS 7 months Median OS 9 months	Neutropenia 71% Febrile neutropenia 40% Sepsis 23%	
Davids et al. [98] NCT03054896	*n* = 27 DLBCL-RT	Phase II of C1 DA-R-EPOCH, followed by venetoclax ramp-up, then venetoclax 400 mg D1-10 of C2-6 of DA-R-EPOCH followed by alloSCT/CAR-T or venetoclax 400 mg daily maintenance	ORR 62% CRR 50%	Median PFS 10 months Median OS 20 months	Neutropenia 58% Anemia 62% Thrombocytopenia 50% Febrile neutropenia 38% Hypophosphatemia 23% Hyponatremia 15% Hyperglycemia 15%	

In the phase 1 first-in-human study of venetoclax in B-NHL, venetoclax achieved an ORR of 75% and CRR of 21% in a cohort of 28 patients with R/R MCL. These patients were heavily pre-treated (median 3 prior lines), but naïve to BTKis [35]. The median PFS and duration of response (DOR) were 11 and 16 months, respectively, with prolonged DOR among the six patients attaining a CR (median 32 months) [38]. Since this formal prospective evaluation, published data on single-agent venetoclax are derived predominantly from retrospective cohorts, as prospective studies have focused on combinations with other targeted therapies, especially BTKis [78].

In a retrospective analysis of 20 patients who received venetoclax for high-risk, BTKi-exposed MCL, response rates were modest (ORR/CRR: 53%/18%) and the median PFS was poor at 3 months [79]. In another retrospective study of 24 patients with high-risk MCL (median 5 prior lines of therapy; 67% BTKi resistant), venetoclax-based regimens (83% monotherapy ± anti-CD20 antibody) similarly achieved an ORR of 50% and CRR of 21%, with a median PFS of 8 months [80]. A multicenter retrospective analysis of 81 patients with R/R MCL (median 3 prior lines of therapy; 91% BTKi-exposed), venetoclax-based regimens (75% monotherapy ± anti-CD20 antibody) achieved an ORR of 42% and CRR of 18%, with a median time on venetoclax of 3 months [81]. Overall, these data suggest that although venetoclax achieves responses in MCL, including some deep remissions, the duration of benefit is short-lived in heavily pre-treated patients, especially those with BTKi-resistant disease. The greatest benefit of venetoclax in MCL is therefore likely to be derived in earlier lines of therapy, or in combination with other targeted agents. In an ongoing phase II study in elderly patients with MCL, all patients received initial induction with rituximab plus bendamustine–cytarabine, but high-risk patients (Ki67 ≥ 30%, blastoid morphology, *TP53* aberrant) receive an abbreviated chemotherapy course followed by venetoclax consolidation and maintenance [82]. Data from this study are awaited with interest and may represent a novel strategy of risk-adapted incorporation of venetoclax in conjunction with chemo-immunotherapy.

### Follicular lymphoma

BCL2 is overexpressed in FL via the canonical chromosomal translocation, t(14;18), establishing a central role in disease biology and an attractive therapeutic target [83]. Clinical trials investigating venetoclax monotherapy or combined with anti-CD20 monoclonal antibodies or chemotherapy are summarized in Table 4. Venetoclax monotherapy achieved modest responses among 29 patients with heavily pre-treated FL in the first-in-human phase I study (ORR 38%; CR 17%), with a median PFS and DOR of 11 and 27 months, respectively. As seen in CLL and MCL, greater response depth was associated with prolonged DOR (PR: median DOR 10 months; CR: median DOR 38 months) [35, 38]. Higher response rates were observed in cohorts assigned doses ≥ 900 mg daily, with an ORR of 44% among those assigned 1200 mg daily compared to 13% among those receiving ≤ 600 mg daily, suggesting a dose–response relationship. Although promising, the modest single-agent efficacy suggested that combination with chemotherapy or other novel agents would be required to improve outcomes. A phase I study of 25 treatment-naïve patients investigated doses of venetoclax 600-800 mg daily combined with obinutuzumab for six cycles, followed by 2 years of obinutuzumab maintenance among patients with disease response. Among the 11 response-evaluable patients, the ORR/CRR was 82%/45% with two instances of PD. Grade III/IV toxicities to date have been predominantly hematological (neutropenia 28%, febrile neutropenia 8%), with one discontinuation due to thrombocytopenia, hepatic enzyme rise and pneumonitis [84]. Although preliminary, these response rates appear comparable to those after obinutuzumab–chemotherapy for frontline management of FL in the GALLIUM study (ORR/CRR: 89%/20%)[85].

Based on preclinical evidence of synergy [9], a phase Ib study investigated venetoclax combined with BR in patients with B-NHL, including 32 patients with R/R FL. In the FL cohort, the regimen achieved an ORR of 75% and CRR of 38%, and the median PFS had not been reached at the time of the reporting, although follow-up was short. Across the overall cohort, grade III/IV neutropenia occurred in 60%, although febrile neutropenia was rare (8%). A maximum tolerated dose (MTD) of venetoclax in combination with BR was not reached, and the investigators concluded that 800 mg daily was recommended for phase II trials [86]. The similarly designed phase Ib CAVALLI study examined the combination of venetoclax with CHOP (cyclophosphamide, doxorubicin, vincristine and prednisolone) with rituximab or obinutuzumab. Grade III/IV neutropenia and febrile neutropenia were common (54% and 33%, respectively), requiring protocol adjustments to deliver venetoclax over a fixed period (C1D4-10 and D1-10 for subsequent cycles) rather than continuously. Nevertheless, disease responses among the 24 patients with FL (20 treatment-naïve; 4 R/R) were robust, with an ORR of 83%, CRR of 75% and a 12-month PFS between 90–100% across the rituximab- and obinutuzumab-treated cohorts [87]. These results compared favorably to the R-CHOP arm of the contemporaneous phase III REVELANCE study (R-CHOP vs R-lenalidomide in patients with treatment-naïve FL), which reported an ORR of 65%, CRR of 53% and an estimated 3-year PFS rate of 78% [88]. In another recently reported phase II study, the addition of venetoclax to frontline bendamustine–obinutuzumab in patients with high tumor burden FL achieved a CRR of 73%; however, serious AEs were common (56%), particularly opportunistic infections, inferring that this combination is unacceptably immunosuppressive [89]. Within the limitations of cross-trial comparisons, these data suggest that the addition of venetoclax to standard chemotherapy regimens may improve response rates, although likely at the expense of increased rates of myelosuppression, immunosuppression and infectious complications.

The best data currently available to inform the potential utility of adding venetoclax to conventional chemotherapy in FL comes from the randomized phase II CONTRALTO study, in which patients with R/R FL were assigned at investigator discretion to receive either 1 year of venetoclax plus rituximab (arm A, *n* = 52) or a chemotherapy-containing regimen. Patients in the chemotherapy-containing cohort were randomized 1:1 to receive six cycles BR with 1 year of venetoclax (arm B, *n* = 51) or without (arm C, *n* = 51). While the response rates in the BR cohorts were similar with or without venetoclax (ORR 84% CRR 69–75%), responses were less frequent in the venetoclax–rituximab cohort (ORR 35% CRR 17%). These response data correlated with PFS outcomes, with no difference in the BR cohorts with or without venetoclax (estimated 18-month PFS 62% vs 59%), but significantly inferior outcomes in the venetoclax–rituximab arm (estimated 18-month PFS 27%). Furthermore, toxicity was increased with the addition of venetoclax to BR (grade III/IV neutropenia: 59% vs 28%; febrile neutropenia 12% vs 6%) and dose reductions and treatment discontinuations were more common. In somewhat disappointing contrast to CLL/SLL, this non-randomized study demonstrated inferior efficacy with venetoclax–rituximab compared to conventional chemotherapy in R/R FL, and increased toxicity without improved efficacy when venetoclax was added to BR [90].

Overall, venetoclax is active in FL and can achieve CRs in a subset of patients. Despite the high prevalence of t(14;18) and the associated overexpression of BCL2 seen in FL, clinical data have demonstrated inferior efficacy with BCL2 inhibition compared to CLL/SLL or MCL. Although an abbreviated dosing schedule may reduce toxicities and dose interruptions when venetoclax is added to chemo-immunotherapy, the lack of evidence for increased efficacy over conventional approaches casts doubt over this strategy. Future success with venetoclax for patients with this disease will require better selection of patients with BCL2 dependent biology or more efficacious combinations with reduced likelihood of synergistic myelosuppression, preferentially with targeted agents.

### Diffuse large B cell lymphoma and other aggressive B cell lymphomas

Venetoclax monotherapy has limited efficacy in R/R DLBCL, achieving an ORR of 18% (CRR 12%) and median PFS of 1 month among 34 patients in the phase I study [35]. These results suggested that venetoclax is active in some patients with DLBCL, but combination therapy or biomarker identification of likely responders would be required to improve clinical utility. For example, BH3 profiling of primary aggressive B-NHL cells demonstrates that approximately one-third harbor defects in pro-apoptotic proteins such as BAX, BAK, BIM or BID that confer venetoclax resistance [91]. Although further validation is required, these assays may ultimately prove useful clinical tools to predict response to BH3-mimetic drugs. In the phase 1b study of venetoclax plus BR, the ORR among 22 patients with R/R DLBCL was 41% (CRR 14%), with poor PFS (median 4 months) [86]. In the phase 1b CAVALLI study, outcomes among 18 patients with treatment-naïve DLBCL were more promising when venetoclax was added to rituximab/obinutuzumab-CHOP, with a CRR of 89% and an estimated 12-month PFS rate between 70 and 100%. Interestingly, CRs were achieved among seven out of eight patients with double-expressor status, and three out of four patients with MYC rearrangements by fluorescent in situ hybridization (FISH), suggesting that the addition of venetoclax may improve efficacy within these higher-risk subsets [87].

These results led to the larger phase II CAVALLI study of 206 patients with treatment-naïve DLBCL and an international prognostic index (IPI) of 2–5. The R-CHOP arm of the contemporaneous GOYA study (rituximab vs obinutuzumab plus CHOP in frontline DLBCL) was used as a pre-specified covariate-adjusted historical control [92]. Patients received CHOP for 6 cycles, with the option for a further 2 cycles at investigator discretion (utilized in < 10% of patients), with venetoclax 800 mg on C1 D4-10 and D1-10 for cycles 2–8 and standard rituximab. The patients were similar to the IPI 2–5 R-CHOP cohort of GOYA across baseline characteristics, although elevated BCL2 expression by immunohistochemistry was more prevalent in the CAVALLI cohort (58% vs 49%). Six or more cycles of R-CHOP were completed in 74% of patients, and 70% completed the planned venetoclax treatment. Relative dose intensity for CHOP was similar across the cohorts, although rituximab intensity was reduced among venetoclax-treated patients due to dose delays. Although response rates were comparable between groups, the CAVALLI cohort demonstrated significantly superior PFS compared to historical controls in the overall population (hazard ratio [HR] 0.61 95%CI [0.43–0.87]) and particularly among patients with high BCL2 expression by immunohistochemistry (HR 0.55 95%CI [0.34–0.89]). The 2-year OS was 86% in the overall CAVALLI cohort and 81% in the covariate-adjusted GOYA R-CHOP control, without a statistically significant difference (HR 0.72 95%CI [0.48–1.10]). Toxicities were more common among venetoclax-treated patients, with higher rates of grade III/IV AEs (86% vs 66%) and serious AEs (56% vs 41%), which were predominantly related to myelosuppression and febrile neutropenia. There was no increase in fatal AEs (2% in CAVALLI vs 5% in GOYA controls)[93]. Overall, these data suggest that venetoclax can be added to R-CHOP without compromising dose intensity, although hematologic and infectious complications are likely increased. The suggestion of improved efficacy, particularly among patients with BCL2 overexpression by immunohistochemistry, is promising and requires formal study in randomized trials, which are currently active (NCT03984448).

A recently published phase I study reported the safety and preliminary efficacy of venetoclax added to six cycles of dose-adjusted-R-EPOCH (etoposide, prednisolone, vincristine, cyclophosphamide and doxorubicin) in 30 patients with treatment-naïve aggressive B cell lymphoid neoplasms (50% with BCL2 and MYC rearrangements by FISH, “double hit lymphoma” [DHL]). Although unacceptable dose-limiting toxicities were not observed at the maximum dose level (800 mg for 10 days per cycle), the investigators selected 600 mg for 5 days as the recommended phase II dose (RP2D) in combination with R-EPOCH due to improved overall tolerability and reduced duration of cytopenias to facilitate maintenance of chemotherapy dose intensity. Venetoclax dose reductions and discontinuations were required in five patients (17%), and 30% of patients discontinued treatment due to toxicity. Grade III/IV hematologic complications were common (neutropenia, 83%; thrombocytopenia, 70%; anemia, 60%) and 63% of patients experienced febrile neutropenia. There was one septic death, one instance of intracranial hemorrhage and eight instances of serious gastrointestinal complications including ileus, colonic perforation and bowel obstruction. Although toxicities were common, 93% of patients achieved a CR and the estimated 24-month PFS was 83%, representing impressive efficacy in this high-risk cohort. Among patients with DHL, 87% attained a CR and the estimated 24-month PFS was 65%. Overall, this study suggested that the addition of venetoclax to R-EPOCH is associated with a high rate of toxicities and likely compromises chemotherapy dose intensification, although this may be offset by potentially improved outcomes among high-risk populations for whom current standard regimens are associated with poor outcomes [94]. Disappointingly, Alliance 051701, a randomized study of venetoclax plus R-EPOCH for patients with double-hit or double-expressor DLBCL, was terminated early due to excess deaths (four due to sepsis, two cardiac arrests) in the venetoclax plus R-EPOCH arm [95]. Despite initial promise, the combination of venetoclax with an intensive chemotherapy regimen such as EPOCH appears to be unacceptably toxic.

Overall, current data suggest that the addition of venetoclax to standard chemo-immunotherapy regimens in aggressive B cell lymphomas has the potential to improve outcomes for some patients, but likely at the expense of increased neutropenia and infectious complications (summarized in Table 4). The combination of venetoclax with R-EPOCH is excessively toxic, whereas the balance of efficacy and toxicity when added to R-COP may be more favorable. Evaluation of this strategy in randomized trials is clearly needed, as are biomarkers of BCL2 dependence, which will ideally enable identification of patients most likely to benefit [96]. Progress in the biological subtyping of DLBCL by molecular profiling may also identify patients whose disease is most vulnerable to BCL2 inhibition and warrants dedicated study in future clinical trials [97].

### Richter transformation

In contrast to CLL/SLL, evidence to inform use of venetoclax to treat Richter transformation (RT) is scarce. Seven patients with DLBCL-type RT received venetoclax monotherapy in the first-in-human phase 1 study, three of whom achieved a partial response (PR) [35]. Preliminary data are available from a phase II study in which 26 patients received a cycle of dose-adjusted-R-EPOCH, followed by accelerated inpatient venetoclax ramp-up and subsequently venetoclax 400 mg daily on D1-10 of the remaining five R-EPOCH cycles. Patients achieving objective disease response were planned to proceed to allogeneic stem cell transplantation (alloSCT), chimeric antigen receptor T cell (CAR-T) therapy, or venetoclax 400 mg daily maintenance. The ORR was 62% and CRR was 50%, with a median PFS and OS of 10 and 20 months, respectively. Of the 18 candidates for cellular therapy, nine (50%) have proceeded to alloSCT or CAR-T [98]. Consistent with other trials combining venetoclax with intensive chemotherapy, hematologic toxicity and frequent febrile neutropenia (38%) were reported, with no instances of TLS. Given the dismal historical outcomes of patients with RT [99, 100], these results are highly promising; however considerable caution is required given the excess mortality observed in the Alliance A051701 trial. Although longer-term follow-up and evaluation in randomized studies are awaited, this preliminary evidence of efficacy suggests that additional of venetoclax to chemo-immunotherapy may improve outcomes in this high-risk population for whom improved therapies are desperately needed. Careful consideration to venetoclax dosing and scheduling, the intensity of the chemotherapy backbone and monitoring for excess toxicity will be critical in future trials.

### Waldenstrom macroglobulinemia

Gene expression analyses have identified that BCL2 is frequently upregulated in Waldenstrom macroglobulinemia, a transcriptional profile more closely resembling CLL than multiple myeloma [101, 102]. Consistent with this preclinical evidence of a pathobiological role of BCL2, all four patients with Waldenstrom macroglobulinemia who received venetoclax in the phase 1 study in patients with B-NHL attained a PR, with a median PFS and DOR 30 and 25 months, respectively [35, 38]. In a phase II study of venetoclax monotherapy 800 mg daily for two years in 32 patients with RR Waldenstrom macroglobulinemia (median 2 prior lines of therapy; 50% BTKi-exposed; *CXCR4* mutations 53%), the ORR was 84% with a major response rate of 81% and median PFS of 30 months. Progression events were frequent in the period immediately following venetoclax cessation, suggesting that continuous therapy may achieve more durable disease control. The major toxicity was neutropenia (44% grade III/IV) [103]. These results suggest significant clinical activity in Waldenstrom macroglobulinemia, and trials investigating venetoclax combination with other targeted agents are currently active (NCT04840602).

### Marginal zone lymphoma

Data specifically pertaining to patients with marginal zone lymphoma (MZL) are limited. Among the three patients who received venetoclax in the phase 1 study in B-NHL, two PRs were observed, with a median PFS and DOR of 21 and 20 months, respectively [35, 38]. Six patients with R/R MZL received venetoclax-BR in the phase 1b study, achieving an ORR of 100% and CRR of 50%. The median PFS and DOR were 12 and 10 months, respectively [86]. As seen with other therapeutic advances, dedicated analyses of this indolent subtype are made difficult by their relatively rarity and insights are largely extrapolated from broader cohorts of B cell malignancies [104].

### Acute lymphoblastic leukemia/lymphoma

Based on preclinical evidence of dependence on BCL2 and BCL-X_L_ [105–107], several small series have investigated the use of BH3-mimetics in acute lymphoblastic leukemia/lymphoma (ALL). In an early report of a phase I study of 18 predominantly older adults with Philadelphia chromosome negative (Ph-)ALL (56% treatment-naïve), escalating doses of venetoclax (400–600 mg daily) were added to mini-hyper-CVD/MA (cyclophosphamide, vincristine, dexamethasone, alternating with cycles of methotrexate and cytarabine) followed by alloSCT or venetoclax plus POMP (6-mercaptopurine, vincristine, methotrexate and prednisolone) maintenance. The most common grade III/IV AE was febrile neutropenia (39%). Among the ten patients with treatment-naïve ALL, the ORR/CRR was 100%/90%, with uMRD by flow cytometry in all instances of CR. Six patients proceed to alloSCT with no disease relapse over a median follow-up of 11 months. Among the eight patients with R/R disease, 38% achieved a CR and 25% attained uMRD, with two ongoing responses up to ~ 4 months. A recently reported phase II study also combined venetoclax with mini-hyper-CVAD/MA in elderly patients with untreated Ph-ALL (*n* = 4) or adult ≥ 18 years old with relapsed disease (*n* = 19). Nelarabine and PEG-asparaginase were incorporated into consolidation and maintenance for patients with T cell ALL, and all patients were intended to receive maintenance vincristine, prednisolone and venetoclax or proceed to alloSCT. Among the four frontline patients, three attained MRD-negativity and all remain in remission at a median follow up of 1 year (one proceeded to alloSCT). Among the relapsed cohort, 65% attained CR, three patients attained MRD-negativity and one proceeded to alloSCT, with a median PFS and OS of 6 and 7 months, respectively [108]. In a retrospective analysis of patients with R/R T-ALL, 13 patients received venetoclax with chemotherapy and 60% of evaluable patients achieved a morphological CR, although the median OS was short (8 months) [109]. In another retrospective series, 18 pediatric and adolescent/young adult patients received venetoclax for relapsed ALL/lymphoblastic lymphoma (T cell, *n* = 13; B cell *n* = 5), with a similar CRR of 61% and short survival (median OS 9 months) [110]. In the largest published prospective study to date, 47 pediatric and adult patients with R/R ALL (40% T cell ALL) received escalating doses of navitoclax (25-100 mg daily) in combination with venetoclax 400 mg daily and chemotherapy/asparaginase. CRs were achieved in 60% of patients, with comparable results across B cell, T cell, adult and pediatric subgroups. BM uMRD was attained in 34% of patients, and 28% of subjects proceeded to alloSCT or CAR-T therapy. Grade III/IV toxicities were predominantly hematological, including febrile neutropenia in 47% of patients. Correlative BH3 profiling confirmed frequent BCL2 and BCL-X_L_ dependency in ALL cells, and the capacity for dependency switching during treatment as a potential mechanism of resistance to BCL2 inhibition [50]. Overall, these data suggest that BH3-mimetics have activity in ALL and achieve deep responses, although these are frequently short-lived and should be utilized to facilitate cellular therapies in the R/R setting. These are particularly important in the context of T-ALL, where effective therapeutic options are limited for patients with R/R disease [111], and for older patients, for whom currently modalities are frequently unacceptably toxic or do not achieve durable control [112].

### Multiple myeloma

Myeloma cell lines and primary cells exhibit variable BCL2 family member expression and dependency, and in vitro venetoclax sensitivity in myeloma cells correlates to the relative expression of alternative pro-survival proteins, particularly MCL1 [113–115]. Primary cells from patients whose myeloma harbored t(11;14) exhibited marked venetoclax sensitivity compared to other molecular subsets [116], positioning this cytogenetic lesion as a biomarker of interest in clinical trials. Venetoclax sensitivity does not appear to be dependent on this translocation, however, and this observation may be driven by enrichment for B cell like transcriptional and epigenetic characteristics among this subset, with associated increased dependence on BCL2 [117]. Furthermore, there is preclinical evidence of synergy between venetoclax and conventional anti-myeloma therapies such as dexamethasone and proteasome inhibitors, which have been shown to upregulate BIM and the MCL1 selective BH3-only protein NOXA, respectively, thereby increasing dependence on BCL2 in myeloma cell lines and primary cells [118–121].

Clinical trials evaluating venetoclax in multiple myeloma are summarized in Table 5. In a phase 1 dose escalation study of venetoclax in heavily pre-treated patients (median number of prior therapies 5; 61% bortezomib and lenalidomide refractory), the ORR was 21%, with 15% of patients attaining a very good partial response or better (≥ VGPR). Responses were more frequent among the 30 patients with disease positive for t(11;14), with an ORR of 40% and ≥ VGPR rate of 27%. Although sustained remissions up to 2 years occurred in rare patients, the overall cohort time to progression (TTP) was 2.6 months. Toxicities were most commonly hematological and serious infectious AEs (sepsis, pneumonia) occurred in 13% of patients, although doses up to 1200 mg daily were generally well tolerated. A high ratio of BCL2/BCL-X_L_ expression by digital droplet polymerase chain reaction (PCR) in CD138-enriched BM mononuclear cells was associated with superior frequency, depth and duration of response and was enriched among the t(11;14) harboring subset [122].

**Table 5:** Studies of BH3-mimetics in multiple myeloma

Study	Cohort	Design	ORR/ ≥ VGPR	PFS/OS	III/IV toxicity (> 10%)	Note
*Phase I/II*						
Kumar et al.[122] NCT01794520	*n* = 66 R/R myeloma, median 5 prior lines of therapy 46% t(11;14)	Phase 1 dose escalation venetoclax monotherapy 300-1200 mg daily	ORR 21% ≥ VGPR 15% t(11;14): ORR 40% ≥ VGPR 27%	Median TTP 2.6 months t(11;14) median TTP 6.6 months	Thrombocytopenia 26% Neutropenia 21% Anemia 14% Lymphopenia 15%	
Kaufman et al. [123] NCT01794520	Phase 1: *n* = 20 (median 3 prior lines of therapy) Phase 2: *n* = 31 (median 5 prior lines of therapy) RR myeloma with t(11;14)	Phase 1/2 study of venetoclax 800 mg daily plus 20-40 mg dexamethasone on D1, 8 and 15	Phase 1: ORR 60% ≥ VGPR 30% Phase 2: ORR 48% ≥ VGPR 36%	Phase 1: Median TTP 12.4 months Phase 2: Median TTP 10.8 months	Lymphopenia 20% Anemia 12% Neutropenia 10% Thrombocytopenia 10%	
Moreau et al. [124] NCT01794507	*n* = 66 R/R myeloma Median of 3 prior lines of therapy 14% t(11;14)	Phase 1b study of venetoclax 50-1200 mg daily plus bortezomib and dexamethasone	ORR 67% ≥ VGPR 42%	Median TTP 9.5 months	Thrombocytopenia 29% Anemia 15% Neutropenia 14%	RP2D in combination with bortezomib and dexamethasone 800 mg daily
Bahlis et al. [126] NCT03314181	Part 1: R/R myeloma with t(11;14), *n* = 24 Part 2: RR myeloma (cytogenetically unselected), *n* = 24	Phase 1 study of venetoclax 400-800 mg daily plus: Part 1: Daratumumab plus dexamethasone Part 2: Daratumumab, bortezomib and dexamethasone	Part 1: ORR 96% ≥ VGPR 96% Part 2:ORR 92% ≥ VGPR 79%	Part 1: 18-month PFS 91% Part 2: 18-month PFS 67%	Part 1: Neutropenia (21%) Hypertension (17%) Part 2: Thrombocytopenia (17%) Lymphopenia (13%) Insomnia 25%	One death due to sepsis in part 2 (in context of progressive disease)
Costa et al.[125] NCT02899052	*n* = 49 R/R myeloma (median 1 prior line of therapy)	Phase 2 study of venetoclax 400 or 800 mg plus carfilzomib and dexamethasone	ORR 80% ≥ VGPR 65% t(11;14) ORR 92% ≥ VGPR 85%	Median PFS 27 months	Lymphopenia 31% Hypertension 16% Pneumonia 12% Neutropenia 12% Hypophosphatemia 10% Diarrhea 10% Insomnia 10%	
*Phase III*						
BELLINI [130, 131] NCT02755597	R/R myeloma, 1–3 prior therapies *n* = 97	Phase III randomized controlled trial of bortezomib and dexamethasone plus: Placebo	ORR 68% ≥ VGPR 36% t(11;14) ORR 47% ≥ VGPR 27%	Median PFS 12 months 36 (37%) deaths at median follow up 46 months	Diarrhea 11% Thrombocytopenia 30% Anemia 15%	Increased mortality in venetoclax arm, predominantly due to infections
	*n* = 194	Venetoclax 800 mg daily	ORR 82% ≥ VGPR 59% t(11;14) ORR 90% ≥ VGPR 70%	Median PFS 23 months 78 (40%) deaths at median follow up 46 months	Pneumonia 16% Diarrhea 15% Thrombocytopenia 15% Anemia 15% Neutropenia 18%	

In a phase 1/2 study of venetoclax 800 mg daily plus dexamethasone in patients with R/R myeloma with t(11;14), the ORR was 48–60%, with ≥ VGPR in 30–36% of patients, and the median TTP was 11–12 months. Baseline BCL2 expression by quantitative PCR in CD138-enriched BM mononuclear cells was associated with response [123]. In a phase 1b study, escalating doses of venetoclax were combined with bortezomib and dexamethasone in a cohort of patients with R/R myeloma (median 3 prior lines of therapy, 14% t(11;14)). Toxicities were again predominantly hematological and no MTD was reached. The ORR was 67% (≥ VGPR 42%) with a median TTP of 9.5 months, with expectedly superior responses and duration of benefit among the non-bortezomib refractory subset (ORR 90% ≥ VGPR 64%; median TTP 11.3 months). Again, higher BM BCL2 expression was associated with response. The RP2D of venetoclax in combination with bortezomib–dexamethasone of 800 mg daily was selected based on frequent grade III/IV neutropenia at doses > 800 mg daily [124].

In a recent phase II study, the combination of venetoclax with carfilzomib and dexamethasone also demonstrated safety and preliminary efficacy, including frequent deep responses among patients with t(11;14) (≥ VGPR 85%) [125]. In a small phase 1 study, venetoclax was combined with daratumumab and dexamethasone in a cohort of patients with t(11;14) positive R/R myeloma, achieving ≥ VGPR in 96% of patients, with an estimated 18-month PFS of 91%. In a separate arm of the study, venetoclax was combined with daratumumab, bortezomib and dexamethasone for the treatment of 24 cytogenetically unselected patients with R/R myeloma, and achieved ≥ VGPR in 79% of patients, with an estimated 18-month PFS of 67% [126].

Overall, these datasets confirm that venetoclax-based regimens are active in myeloma, with suggestion of particular benefit among patients with t(11;14) positive disease or elevated BCL2 expression. Retrospective “real world” datasets also support the efficacy of venetoclax in t(11;14) positive myeloma, with frequent responses (> 90%) and a median PFS of 10 months among patients with R/R disease in one series [127]. Responses have also been observed among patients with plasma cell leukemia, in which t(11;14) is frequently detectable, and future studies are required to further evaluate the role of BCL2 inhibition is this high-risk disease [127–129].

In the context of these early phase studies, the randomized phase III BELLINI trial was performed, in which 291 patients with myeloma previously treated with 1–3 lines of therapy were randomized 2:1 to receive bortezomib–dexamethasone plus venetoclax or placebo. Responses and PFS were superior among the venetoclax cohort; however, there was increased mortality due to treatment-emergent fatal infections (eight in the venetoclax arm, zero in the placebo arm), mostly following disease progression [130, 131]. Although these results for the overall cohort were disappointing, patients with disease positive for t(11;14) or high BCL2 expression had particular benefit in terms of depth of response (MRD 10^–5^:19% in venetoclax cohort vs 0% in placebo) and PFS (median PFS 36.8 vs 9.3 months, respectively), without the excess mortality observed in the overall group, although this may be due to reduced power [130–132]. Further studies are required to determine whether the benefit-toxicity balance will be more favorable among these subgroups. There are limited data from the BELLINI study on the immunosuppressive effects of venetoclax in patients with myeloma. Data from the phase I M15-654 study evaluating lymphocyte subsets following venetoclax-based therapies identified early and profound suppression of normal B and CD4 + T cells, with transient effects on CD8 + T cell numbers [126, 133]. The investigators also described an increase in T cell clonality/loss of T cell clonal richness. Further studies are required to further understand the complex immune effects of venetoclax in myeloma, including the immunosuppression leading to increased rates of serious infections and the differential effects on T cell subsets, such as naïve T vs memory T cells, which may promote anti-tumor T cell responses [134, 135]. Similar to data from patients with aggressive B-NHL, the results of these clinical trials suggest that the activity of venetoclax in myeloma must be carefully balanced against immunosuppression and infectious toxicities. It seems likely that biomarker-driven selection of sensitive disease subtypes will be critical to maximizing the potential of BH3-mimetics in this setting.

### Lessons from venetoclax in lymphoid neoplasms other than CLL/SLL

Venetoclax has single-agent activity in a broad range of lymphoid neoplasms, with MCL and Waldenstrom macroglobulinemia demonstrating the greatest intrinsic sensitivity to BCL2 inhibition. In these two diseases, combination regimens with anti-CD20 monoclonal antibodies or other targeted agents are likely to be potent and may challenge current standard of care regimens in the near future (e.g., NCT03112174, NCT04273139 and NCT03523975). In FL, DLBCL and multiple myeloma, responses are less consistent; however, subsets of patients can achieve deep and durable remissions. Further research to prospectively identify patients with sensitive disease may improve the clinical utility of BH3-mimetics, a strategy which is most advanced in multiple myeloma, where t(11;14) and elevated BCL2 expression have been consistency associated with superior responses. The addition of venetoclax to conventional chemotherapy or standard anti-myeloma regimens results in synergistic myelosuppression and infection risk, which may be attenuated through abbreviated courses of venetoclax instead of continuous dosing in some settings. In particular, the combination of venetoclax with DA-R-EPOCH, bendamustine–obinutuzumab and bortezomib–dexamethasone has been associated with excessive toxicity and highlights critical safety signals to be closely monitored in future trials combining venetoclax with conventional treatments. Despite these caveats, preliminary data suggest that the addition of venetoclax to conventional chemotherapy may improve response rates among high-risk neoplasms with historically poor outcomes, such as RT, DHL and high-risk ALL. Randomized studies are required to confirm the preliminary evidence of improved outcomes with venetoclax-R-CHOP observed in the CAVALLI study and venetoclax-based combinations in multiple myeloma with t(11;14) positivity and high BCL2 expression.

## Associations and mechanisms of resistance to pro-apoptotic agents

Our current understanding of the mechanisms of resistance to BH3-mimetics is predominantly derived from cohorts of patients with heavily pre-treated R/R CLL receiving continuous venetoclax monotherapy. While critical insights have been made, their relevance to patients receiving frontline fixed-duration combination regimens is uncertain, as are their implications for other disease types. In the context of indefinite venetoclax monotherapy for R/R CLL, the clinicopathological factors associated with inferior PFS on multivariate analysis include disease bulk (≥ 5 cm), BCRi or fludarabine refractoriness, *TP53* aberrations and *NOTCH1* mutations [44]. Among patients who received venetoclax–rituximab for R/R CLL in the MURANO study, high genomic complexity was associated with inferior PFS, and lower uMRD rates were observed among patients with disease harboring *TP53, NOTCH1, BRAF* and *BIRC3* mutations [64]. Furthermore, among patients who attained uMRD, del(17p), genomic complexity or IGHV-unmutated status was associated accelerated MRD recrudescence and shorter time to PD [62]. On multivariate analysis of the venetoclax–obinutuzumab cohort of CLL14, only del(17p) was associated with inferior PFS [136]. Overall, although patients with traditional risk factors benefit most from the use of venetoclax-based regimens instead of chemo-immunotherapy, they nevertheless retain an adverse prognosis, especially those with *TP53* aberrant disease. Consistent with these clinical observations, in vitro experiments suggest that although venetoclax is active against p53 perturbed CLL [137], these lesions facilitate survival and the evolution of resistance with prolonged sublethal exposure to BCL2 inhibition [138].

The best characterized biological mechanisms of venetoclax resistance in CLL/SLL are acquisition of *BCL2* resistance mutations and upregulation of alternative pro-survival proteins such as BCL-X_L_ and MCL1. Expansion of clones with genomic instability and other genetic mutations have been described among patients with early progression and RT on venetoclax, although their precise role in resistant disease biology requires further study [139, 140]. The first described *BCL2* mutation, Gly101Val, was detected among patients with prolonged venetoclax exposure (median 36 months) and impairs venetoclax binding to the alpha-helical groove of BCL2 without compromising its affinity for BH3-only proteins [48]. Several *BCL2* resistance mutations have now been identified, including in patients with FL and MCL, and have not been detected in pre-treatment samples [141–145]. Interestingly, distinct mutations frequently co-occur within individuals and are present in a highly variable proportion of any single patient’s CLL cell population (< 1 to 83%), implying that multiple resistance mechanisms likely coexist within the leukemic population [142]. Supporting this hypothesis, distinct subclones with wild-type *BCL2* but overexpression of BCL-X_L_ and MCL1 have been found to coexist separately alongside *BCL2-*mutated populations within individual patients [48, 142]. Alternative pro-survival proteins may also be upregulated by the lymph node microenvironment to confer resistance, and in vitro evidence suggests BCRis may disrupt this mechanism [146]. Upregulation of BCL-X_L_ has also been implicated in resistance to venetoclax–ibrutinib therapy for MCL, where chromosome 9p21.1–p24.3 loss and/or mutations in components of the SWI–SNF chromatin-remodeling complex were identified in all patients with primary resistance and two-thirds of patients with relapsed disease. The consequence of these abnormalities was upregulation of BCL-X_L_ transcription, and concomitant BCL-X_L_ inhibition restored cytotoxicity [47]. Other described resistance mechanisms in patients with CLL/SLL include chromosome 1q23 amplification and overexpression of MCL1, with associated changes to mitochondrial metabolism [139]. Among eight patients with early PD and frequent RT, whole-exome sequencing of identified recurrent mutations in *BTG1* and homozygous deletions of *CDKN2A/B*, *PD-L1* amplification in one case, and a *BRAF* mutation postulated to augment MCL1 expression [140]. Emergence of *TP53* aberrations or cytogenetically complex disease has been frequently observed in patients with PD on venetoclax, further implicating genomic instability in the development of resistance [147, 148]. As previously discussed, primary venetoclax resistance due to defects in other components of the intrinsic death pathway is frequently detectable in primary DLBCL cells, and upregulation of MCL1 is associated with in vitro resistance in primary indolent B-NHL cells [91]. As discussed previously, the ratio of BCL2, BCL-X_L_ and MCL1 expression in myeloma cell lines, primary cells and patient samples in clinical trials has been associated with venetoclax sensitivity, suggesting variable relative dependencies on these pro-survival proteins as a driver of intrinsic resistance [113–115, 122, 123, 132].

Overall, among patients receiving indefinite venetoclax monotherapy for CLL/SLL, resistant disease is typically oligoclonal, with multiple coexisting mechanisms including *BCL2* mutations and upregulation of alternative BCL2 family proteins. Mechanisms may differ among patients with early PD, where expansion of clones with genomic instability is frequently observed. Although resistance mechanisms outside of CLL are less well understood, parallel observations in FL and MCL have been reported (Fig. 1). These observations support the use of time-limited combination regimens which may remove the sustained selection pressure for *BCL2-*mutated subclones, and indeed, no *BCL2* mutations have been detected among patients treated with time-limited therapy to date. Concomitant BTK inhibition may subvert the upregulation of alternative BCL2 family proteins by the tumor microenvironment, and combination trials have demonstrated frequent deep remission in CLL and MCL [29, 78, 149, 150]. Further investigation is ongoing into the clinical development of BCL-X_L_ and MCL1 inhibitors to eradicate venetoclax-resistant subpopulations and hopefully enhance the curative potential of BH3-mimetic-based regimens [24].

## Conclusions

BH3-mimetics represent an exciting novel class of small molecule inhibitors of BCL2 family proteins, which restore apoptosis in malignant cells that depend on these proteins for survival. The orally bioavailable selective BCL2 inhibitor, venetoclax, is the most clinically advanced and only currently approved BH3-mimetic, although ongoing investigation of the BCL2/BCL-X_L_ inhibitor, navitoclax, and other BCL2 family inhibitors continues. The major toxicities of venetoclax include TLS and neutropenia, although these are readily preventable/manageable with established protocols. Venetoclax achieves frequent and deep remissions in CLL/SLL and is now a standard of care regimen in relapsed and frontline disease as fixed-duration combination therapy with anti-CD20 monoclonal antibodies. Venetoclax is also active against other lymphoid malignancies, including several B-NHLs, B cell and T cell ALL and multiple myeloma. Outside CLL/SLL, the most promising efficacy has been observed in patients with MCL and Waldenstrom macroglobulinemia, where venetoclax will likely to be incorporated into potent targeted agent combination regimens in future trials. The addition of venetoclax to conventional therapies may improve disease control in high-risk aggressive B cell neoplasms and R/R multiple myeloma, but this must be balanced with increased risk myelosuppression and serious infection as observed in the BELLINI and Alliance A051701 trials. Synergistic myelosuppression and immunosuppression are critical safety signals to be monitored in future trials adding venetoclax to chemo-immunotherapy and standard anti-myeloma therapy. Identification of venetoclax-sensitive disease subsets may be a promising avenue to improve the ratio of benefit to toxicity for these combinations. Venetoclax resistance can be mediated through *BCL2* mutations, upregulation of alternative BCL2 family members, p53 aberrations and genomic instability or defects in other components of the intrinsic cellular death pathway. Future research will focus on therapeutics targeting other BCL2 family members, clinically validated biomarkers for BH3-mimetic sensitivity and novel combinations to improve efficacy.

## Data Availability

Not applicable.

## Abbreviations

AE: Adverse effects
ALC: Absolute lymphocyte count
ALL: Acute lymphoblastic leukemia/lymphoma
AlloSCT: Allogeneic stem cell transplantation
B-NHL: B cell non-Hodgkin lymphoma
BCRis: B cell receptor signaling inhibitors
BM: Bone marrow
BR: Bendamustine–rituximab
BTKi: Bruton tyrosine kinase inhibitor
CAR-T: Chimeric antigen receptor T cell
CLL/SLL: Chronic lymphocytic leukemia/small lymphocytic lymphoma
CR: Complete response
CRR: Complete response rate
DHL: Double-hit lymphoma
DOR: Duration of response
FCR: Fludarabine, cyclophosphamide and rituximab
FL: Follicular lymphoma
MCL: Mantle cell lymphoma
MTD: Maximum tolerated dose
ORR: Objective response rate
OS: Overall survival
PB: Peripheral blood
PCR: Polymerase chain reaction
PD: Progressive disease
PFS: Progression-free survival
PR: Partial response
R/R: Relapsed and refractory
RP2D: Recommended phase II dose
RT: Richter transformation
TLS: Tumor lysis syndrome
TTP: Time to progression
tMN: Treatment-related myeloid neoplasm
uMRD: Undetectable measurable residual disease
VGPR: Very good partial response
